# The Twenty-fifth Meeting of the A. D. A.

**Published:** 1885-08

**Authors:** 


### THE TWENTY-FIFTH MEETING OF THE A. D. A.

The Minneapolis meeting promises to be one of unusual interest. The chairman of the committee of arrangements, Dr. Crouse, has been unwearied in his efforts to make of it a success, and all the dentists of the north-west are bestirring themselves to see that nothing is lacking that might enhance the comfort of the members, or add to the interest of the meeting. Very low rates have been secured upon the railroads, and unusual attention will be shown those who attend the meeting. Minneapolis is such an interesting point to visit, and there are so many places of interest in its vicinity, that a great deal of pleasure is anticipated, to say nothing of the profit of the sessions.

